# Oral administration of oligo fucoidan improves the survival rate, quality of life, and immunity in patients with lung cancer

**DOI:** 10.29219/fnr.v68.10674

**Published:** 2024-07-03

**Authors:** Tu-Chen Liu, Chia-Ju Shih, Ya-Ling Chiou

**Affiliations:** 1 Department of Chest Medicine, Cheng-Ching General Hospital, Taichung, Republic of China; 2 Department of Nutrition (Master Program), Hungkuang University, Taichung, Taiwan, Republic of China

**Keywords:** non-small cell lung cancer, oligo-fucoidan, survival rate, quality of life, immunity

## Abstract

**Background:**

Lung cancer, the most commonly diagnosed cancer globally, has the highest incidence and mortality rates in Taiwan. It can be divided into two types. Non-small cell lung cancer (NSCLC) accounts for approximately 85% of lung cancers, which is further divided into adenocarcinoma, squamous cell carcinoma, and large cell lung cancer accounting for approximately 40%, 25%, and 15% of NSCLC cases, respectively. Small cell lung cancer accounts for approximately 15% of lung cancers. Early systemic therapy NSCLC was based on chemotherapy, and immunotherapy is currently under development. Fucoidan, from brown seaweed extracts, shows promise in mitigating radiation-induced lung fibrosis in animal studies, suggesting its potential as an adjuvant for radiation therapy-related lung fibrosis in lung cancer patients. However, the clinical utility of such adjuvant therapy in lung cancer treatment remains uncertain. The purpose of this study was to investigate the effects of oral administration of oligo-fucoidan on the survival rate, quality of life, and immunity of patients with lung cancer.

**Methods:**

Subjects with Non-small cell lung cancer aged between 20 and 80 were collected from outpatient clinics, divided into control group (n = 7): conventional therapy and fucoidan group (n = 13): received conventional therapy+ oral supplementation of oligo-fucoidan (550 mg × 4 tablets). Data were collected before the study, at weeks 4, 12, and 24 during the study, and to collect 20 ml of peripheral blood, for analysis biochemical data, liver and kidney function, lymphocyte population, inflammation cytokines, and using EORTC QLQ-C30 questionnaire to assess quality of life.

**Results:**

The survival rates of the subjects in the control and fucoidan groups were 20% and 28.6%, respectively. During the study, patients in the fucoidan group experienced a better quality of life than those in the control group, but this difference lacked statistical significance. Oligo-fucoidan increases the CD19 lymphocyte population. The patients in the fucoidan group also had Lower inflammatory cytokine.

**Conclusion:**

Oligo-fucoidan holds promise as an adjuvant therapy to enhance the survival rate, quality of life, and immune function in patients with lung cancer.

## Popular scientific summary

**Figure:**
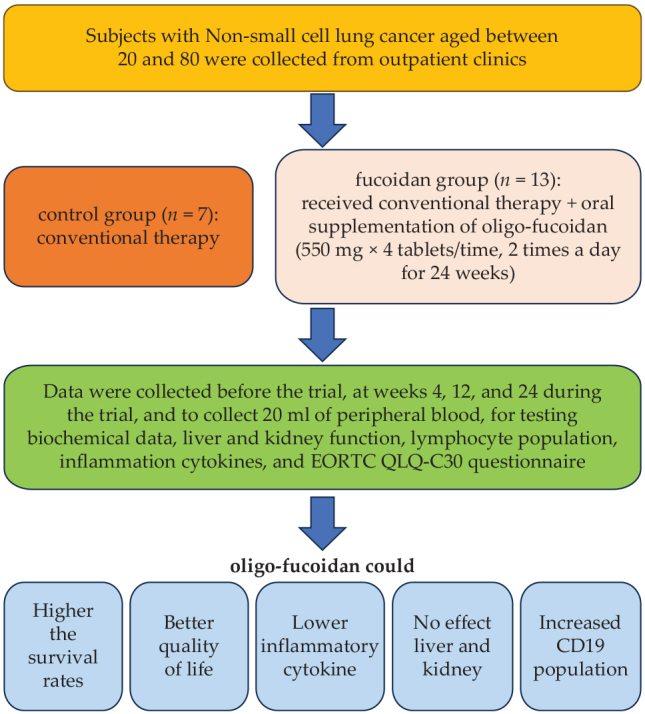


Lung cancer metastasizes easily. In addition, patients with lung cancer have a poor prognosis and high rates of treatment failure and mortality (1). Lung cancer is the most common and deadliest malignant tumour in Taiwan, with the highest incidence and mortality rates (2). Lung cancer is one of the most common cancers worldwide and can be characterized in two types, namely, non-small cell lung cancer (NSCLC, approximately 85% of all lung cancers) and small cell lung cancer (SCLC, less than 15% of all lung cancers). Additionally, three types of NSCLC are known, including adenocarcinoma (approximately 40% of NSCLC cases), squamous cell carcinoma (approximately 25% of NSCLC cases), and large cell lung cancer (approximately 15% of NSCLC cases) (3–5). The above patients with NSCLC were diagnosed at an advanced stage, could not receive surgical radical treatment, and had to rely on systemic therapy. Early systemic therapies for NSCLC were based on chemotherapy, and platinum alloy preparation (cisplatin)-based doublets are currently the standard therapy for lung cancer chemotherapy (6). However, cisplatin is associated with drawbacks, such as side effects and toxicity, including nephrotoxicity, neurotoxicity, nausea and vomiting, and alopecia (7). At present, immune-targeting drugs, such as Nivolumab (Opdivo) (8), Pembrolizumab (Keytruda) (9), and Atezolizumab (Tecentriq) (10) have been developed to activate the immune system of a patient by inhibiting the PD-1 signalling pathway, leading to apoptosis of cancer cells. However, these treatments are associated with fatigue, rash, diarrhoea, mild fever, or inflammatory reactions of the body system, leading to diseases such as hepatitis, gastroenteritis, and interstitial pneumonia. Therefore, the treatment of lung cancer leads to a decline in the quality of life and renders the treatment less efficient, highlighting the need for safer methods with fewer side effects. In recent years, researchers have shown growing interest in readily accessible, naturally occurring compounds due to their potential efficacy in cancer treatment at non-toxic dosages, thereby minimizing or mitigating side effects (11).

Fucoidan is a polysaccharide derived from brown seaweed extracts. It is structurally similar to the heparin molecule. It consists of repeating units of disaccharides containing α-1,3-linked fucose and α-1,4-linked fucose, forming an α-1,3-skeleton. in The C2 position relates to branches (12, 13). Fucoidan exhibits antiviral, antioxidant, antibacterial, anticoagulant, anticancer/antitumor, antiproliferative, and anti-inflammatory effects (13–15). Recent animal experiments indicate that oligo-fucoidan can reduce lung fibrosis induced by radiation therapy for lung cancer by reducing the levels of inflammatory factors. Oligo-fucoidan could be a potential adjuvant used to weaken or prevent lung fibrosis during radiation therapy for lung cancer (16). However, the efficacy of adjuvant therapy for clinical lung cancer treatment is not clear. This study aimed to investigate the effects of oligo-fucoidan as an adjuvant therapy on the survival rate, quality of life, and immunity of patients with NSCLC. This is the first study to publish clinical research on the application of fucoidans in lung cancer. In this study, the survival rate of the fucoidan group (28.6%) was higher than that of the control group (20.0%). In terms of overall quality of life, an increase was noted in the fucoidan group during the study period; the overall quality was improved than that of the control group. Oligo-fucoidan increases the population of CD19 lymphocytes. Neutrophil-to-lymphocyte ratio (NLR) seemed to be lower in the fucoidan group than in the control group. Conclusion: Oligo-fucoidan is a potential adjuvant therapy for improving the survival rate, quality of life, and immunity of lung cancer patients.

## Materials and methods

### Study subjects

The NSCLC subjects were outpatients at the Kuang Tien General Hospital and Cheng Ching Hospital, Chung Kang Branch. The inclusion criteria are listed as follows: (1) Male or female subjects must be aged between 20 and 80 years at the time of screening; (2) The estimated remaining lifespan should exceed 12 weeks, as per the assessment of the test subject; (3) Subjects must be diagnosed with lung cancer based on cytology or histology; (4) During the screening process, the subjects must meet the criteria of being ineligible for surgical resection of lung cancer and have a disease stage beyond or higher than IIIB; (5) In order to meet the requirements of the Response Evaluation Criteria (RECIST version 1.1), the subject must have at least one measurable tumour lesion; (6) The physical condition of the subject must be assessed using ‘East Coast Cancer Research Cooperation Organization (ECOG)’ score, which should be between 0 and 2; (7) Subjects must be able to consume drugs orally; (8) Subjects must meet the following appropriate bone marrow, kidney, and liver functions: (a) Absolute neutrophil count (ANC): 1,500/mm^3^ (1.5 × 10^9^/L); (b) Platelets: 100,000/mm^3^ (100 × 10^9^/L); (c) Haemoglobin: 9.0 g/dL (5.6 mmol/L); (d) Renal function: Serum creatinine (Cre) levels should be within the normal range; (e) Liver function: aspartate aminotransferase (AST) and alanine aminotransferase (ALT) values should be 2.5-fold higher than the upper limit of normal values. Else, the AST and ALT levels should be <5 times the upper limit value and bilirubin levels should be 1.5 times the upper limit of normal value (if the subject has Gilbert’s syndrome, the bilirubin needs to be 3.0 times the upper limit of normal value); (9) The subject must be able to understand the experiment and be willing to follow the instructions of the trial plan and sign the consent form. The exclusion criteria are listed as follows: (1) Grade 2 peripheral neuropathy; (2) Epileptic seizures in the 12 months preceding trial enrolment; (3) Recent use of herbal medicine, over-the-counter anti-cancer supplements, or approved Chinese medicine for tumour treatment within 2 weeks prior to random assignment; (4) Presence of significant uncontrolled clinical symptoms including but not limited to unmanaged nausea, vomiting, diarrhoea, progressive infection, congestive heart failure, unstable angina, arrhythmia, mental illness impacting compliance, history of severe haemoptysis, or any other medical condition posing elevated risk or toxicity, as determined by the moderator; (5) Pregnancy or breastfeeding; (6) Participation in other drug-related clinical trials within 30 days before the screening period.

**Fig. 1:**
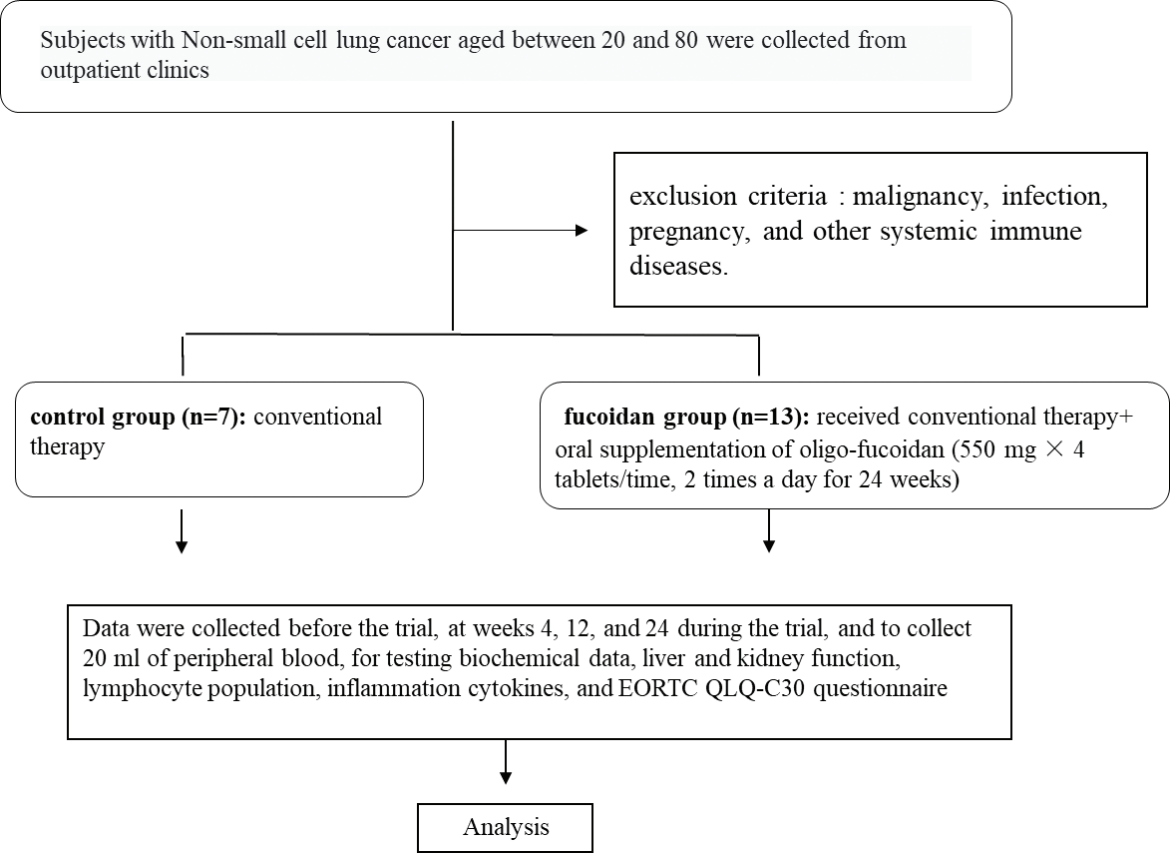
Patient characteristics. Twenty patients were recruited for the study and randomly divided into two groups: (1) control group (*n* = 7), receiving regular treatment and supplementation with placebo, and (2) fucoidan group (*n* = 13), receiving regular treatment and supplementation with oligo-fucoidan (550 mg × 4 tablets at a time twice a day for 48 weeks).

Twenty patients were included in this study and divided into two groups: (1) control group (*n* = 7), which received conventional therapy, and (2) fucoidan group (*n* = 13), which received conventional therapy with oral supplementation of oligo-fucoidan (550 mg × 4 tablets at a time twice a day for 48 weeks) (Fig. 1). All subjects were fully aware of the purpose and nature of the study, which was approved by the Institutional Review Boards (IRB) of Kuang-Tien General Hospital (10802) and Cheng Ching Hospital (HP190023). This study was registered retrospectively, and registration number TCTR20231018001 and the date of registration was 18/10/2023.

## Analysis of basic and biochemical data of the subjects

We collected anthropometric data, including body weight, height, and blood pressure, from the participants using standardized techniques (IRB-approved). Heparinised blood was collected to measure the white blood cell (WBC) count, albumin, pre-albumin, transferrin, T-protein, ALT, AST, T-bilirubin, blood urea nitrogen (BUN), creatinine (Cre), triglyceride (TG), and cholesterol. The remaining blood was separated into serum and cells. Serum aliquots were stored in liquid nitrogen until further analysis.

## Analysis of lymphocyte population

One-third of the total blood cells were incubated with fluorescein isothiocyanate (FITC) anti-human CD19 antibodies. Another third of the total cells were incubated with phycoerythrin (PE)-conjugated anti-human CD3 antibodies for 20 min at 4°C in the dark and were separated and then stained with FITC-conjugated anti-human CD4, anti-human CD8, or anti-human CD25 antibodies for 20 min at 4°C in the dark. The remaining third of the total blood cells were incubated with PE-conjugated anti-human CD4 antibodies for 20 min at 4°C in the dark and stained with FITC-conjugated anti-human CD25, anti-human CD45RA, or anti-human CD45RO antibodies. PE-conjugated mouse IgG1 antibodies were used as isotype controls. Antibodies were purchased from BD Biosciences (Franklin Lakes, NJ, USA).

### Analysis of inflammatory cytokines of the subjects

The plasma were used to analyse the levels of cytokines including, IL-1β, IL-6, and IL-8 using ELISA kits (eBioscience, San Diego, CA, USA). All samples were analysed in duplicate.

## Quality of life assessment

The EORTC QLQ-C30 questionnaire and the National Institute of Health Traditional Chinese Medicine Quality of Life Questionnaire (NHRI QLQ-TCM) are designed to measure cancer patients’ physical, psychological, and social functions, were used for measurements. All scales/items were transformed into scores ranging from 0 to 100. For all scales that measure function, a higher score represented a better Quality of Life, and for all symptom scales/items, a lower score indicated a better Quality of Life.

## Statistical analysis

The results are expressed as the mean ± SD. Statistical Product and Service Solutions (SPSS) for Windows (SPSS Inc., Chicago, IL, USA) was used for statistical analyses. Statistical significance was determined by unpaired *t*-test for comparisons between the control and fucoidan groups and by one-way ANOVA for comparisons between different treatment groups. Two-tailed statistical tests were used, and *P* < 0.05 indicated a statistically significant difference.

## Results

### Patient characteristics

Twenty patients were recruited for the study and randomly divided into two groups: (1) control group (*n* = 7), receiving regular treatment and supplementation with placebo, and (2) fucoidan group (*n* = 13), receiving regular treatment and supplementation with oligo-fucoidan (550 mg × 4 tablets at a time twice a day for 48 weeks) (Fig. 1). The causes of lung cancer and drug therapies are summarized in Table 1.

**Table 1:** The causes of lung cancer and drug therapy

Control group	Fucoidan group
[A-ICD10] C34.32; Therapy: CispIatin 50 mg Taxtere 20 mg	[A-ICD10] C34.91; Therapy: Avastin 100 mg
[A-ICD10] C34.12; Therapy: Filgrastin 300 mg	[A-ICD10] C34.11; Therapy: Cisplatin 50 mg
[A-ICD10] C34.11; Therapy: Taxotere 20 mg	[A-ICD10] C34.12; Therapy: Mepro 500 mg Navelbine 20 mg
[A-ICD10] C34.11; Therapy: Giotrif 30 mg	[A-ICD10] C34.31; Therapy: Avastin 100 mg
[A-ICD10] C34.11; Therapy: Cisplatin 50 mg, Fytosid 100 mg	[A-ICD10] C34.11; Therapy: xalkori 250 mg
[A-ICD10] C34.11; Therapy: Giotrif 30 mg	[A-ICD10] C34.11; Therapy: Giotrif 30 mg
[A-ICD10] C34.11; Therapy: Alimta 500 mg	[A-ICDIO] C34.11; Therapy: Mepro 500 mg
	[A-ICD10] C34.2; Therapy: Xalkori 250 mg
	[A-ICD10] C34.12; Therapy: Keytruda 100 mg
	[A-ICD10] C34.11; Therapy: Cisplatin 50 mg Fytosid 100 mg
	[A-ICD10] C34.12; Therapy: Filgrastin 300 mg
	[A-ICD10] C34.11; Therapy: Alimta 500 mg
	[A-ICDIO] C34.11; Therapy: Avastin 400 mg

ICD-10: The International Statistical Classification of Diseases and Related Health Problems 10th Revision, ICD-10 is a system used by the World Health Organization to classify diseases according to certain characteristics and rules, and to express them using coding methods.

control: no treated Oligo Fucoidan.

Fucoidan group: supplementation with oligo-fucoidan (550 mg × 4 tablets at a time twice a day for 48 weeks).

The basic demographic characteristics of the two groups are shown in Table 2. In terms of nutritional indicators, pre-albumin and transferrin levels tended to decrease in the control group but not in the fucoidan group; however, the difference was not statistically significant (Tables 2 and 3). The examination of the liver function and kidney function indicators showed that fucoidan did not affect the functions of the liver and kidneys (Tables 2 and 3). In addition, NLR was lower in the fucoidan group than in the control group; however, the difference was not statistically significant (Table 2).

**Table 2:** The characteristics of subjects during the supplementation period

Variables	Groups (number)	Baseline	8th week	20th week	36th week	44th week	E-12th week	E 24th week
Age (y/o)	control (*n* = 7)	64.1 ± 13.4	-	-	-	-	-	-
	fucoidan (*n* = 13)	70.9 ± 10.7	-	-	-	-	-	-
Sex (F/M)	control (*n* = 7)	3/4	-	-	-	-	-	-
	fucoidan (*n* = 13)	6/7	-	-	-	-	-	-
BMI (kg/m^2^)	control (*n* = 7)	20.4 ± 3.3	-	-	-	-	-	-
	fucoidan (*n* = 13)	21.9 ± 5.0	-	-	-	-	-	-
WBC (10^3^/uL)	control (*n* = 7)	7.43 ± 19	6.8 ± 2.7	6.7 ± 4.2	-	-	-	-
	fucoidan (*n* = 13)	8.7 ± 4.7	6.1 ± 2.1	7.2 ± 2.1	6.3 ± 1.5	5.5 ± 2.1	4.5 ± 3.5	14 ± 7.1
NLR	control (*n* = 7)	4.8 ± 2.3	4.5 ± 2.1	3.9 ± 2.0	-	-	-	-
	fucoidan (*n* = 13)	14.4 ± 24.6	2.8 ± 1.7	3.9 ± 1.2	5.4 ± 4.7	2.3 ± 0.9	2.3 ± 0.1	18.6 ± 23.3
Albumin	control (*n* = 7)	3.7 ± 0.5	3.6 ± 0.6	4 ± 0	-	-	-	-
	fucoidan (*n* = 13)	3.7 ± 0.5	4 ± 0	3.8 ± 0.4	4 ± 0	4 ± 0	4 ± 0	3.5 ± 0.7
Pre-albumin	control (*n* = 7)	19.6 ± 10.0	13.4 ± 8.5	14.5 ± 5.2	-	-	-	-
	fucoidan (*n* = 13)	17.3 ± 8.2	18.7 ± 8.0	15.8 ± 5.5	16.1 ± 3.2	-	16.5 ± 2.1	26.1 ± 4.3
Transferrin	control (*n* = 7)	195.2 ± 56.6	188.2 ± 47.0	191.7 ± 12.4	-	-	-	-
	fucoidan (*n* = 13)	179.3 ± 56.9	234.4 ± 40.1	201.5 ± 47.0	267.0 ± 19.8	-	247.8 ± 18.7	274.2 ± 25.5
T-Protein	control (*n* = 7)	7.1 ± 0.9	7 0 ± 0.7	7.3 ± 0.6	-	-	-	-
	fucoidan (*n* = 13)	6.6 ± 1.1	7.3 ± 0.5	7.3 ± 0.5	7.5 ± 0.7	7.5 ± 0.7	7.5 ± 0.7	7.0 ± 1.4
GOT (U/L)	control (*n* = 7)	54.7 ± 71.5	29.2 ± 12.2	29.7 ± 18.2	-	-	-	-
	fucoidan (*n* = 13)	29.2 ± 11.7	25.3 ± 9.3	22 ± 4.1	31.3 ± 4.0	24.5 ± 2.1	49.5 ± 33.2*	27.5 ± 13.4
GPT (U/L)	control (*n* = 7)	85.7 ± 174.2	19.2 ± 8.9	17 ± 11.8	-	-	-	-
	fucoidan (*n* = 13)	32.6 ± 24.4	21.9 ± 19.2	17.2 ± 6.9	19.3 ± 7.0	14.0 ± 0	27.5 ± 16.2	16.5 ± 7.8
T- Bilirubin	control (*n* = 7)	7.1 ± 0.9	7.0 ± 0.7	7.3 ± 0.6	-	-	-	-
	fucoidan (*n* = 13)	0.5 ± 0.5	0.3 ± 0.5	0.3 ± 0.5	-	0.5 ± 0.7	0.5 ± 0.7	-
BUN (mg/dl)	control (*n* = 7)	19.9 ± 6.4	17.2 ± 6.0	18.0 ± 9.2	-	-	-	-
	fucoidan (*n* = 13)	20.3 ± 9.2	17.3 ± 5.7	19.0 ± 11.3	12.7 ± 4.2	16.0 ± 5.7	12.0 ± 2.8	20.3 ± 9.2
Creatinine (mg/dl)	control (*n* = 7)	0.8 ± 0.3	0.9 ± 0.2	0.9 ± 0.2	-	-	-	-
	fucoidan (*n* = 13)	0.7 ± 0.2	0.8 ± 0.2	0.8 ± 0.3	0.8 ± 0.3	0.7 ± 0.5	0.7 ± 0.2	0.6 ± 0.1
TG (mg/dl)	control (*n* = 7)	110.6 ± 75.2	-	-	-	-	-	-
	fucoidan (*n* = 13)	123.9 ± 68.4	-	135.4 ± 33.8	114.5 ± 54.5	-	92.0 ± 39.6	79.0 ± 28.3
Cholesterol (mg/dl)	control (*n* = 7)	-	-	-	-	-	-	-
	fucoidan (*n* = 13)	165.9 ± 41.5	-	173.8 ± 38.3	186.5 ± 67.2	-	149.0 ± 7.1	183.5 ± 23.3

BMI = Body Mass Index; WBC = White blood cell; NLR = neutrophil-to-lymphocyte ratio; GOT = aspartate aminotransferase; GPT = alanine aminotransferase; BUN = blood urea nitrogen; TG = Triglyceride; E = during follow period.

* : significant different campared with control group among groups (*P* < 0.05).

**Table 3:** The changed in biochemical data during the supplementation period

Variables	Groups (number)	Δ8th week	Δ20th week	Δ36th week	Δ44th week	ΔE-12th week	ΔE-24th week
WBC (103/uL)	control (*n* = 7)	0.2 ± 2.59	0.67 ± 4.04	-	-	-	-
	fucoidan (*n* = 13)	0 ± 2.31	1 ± 2	1 ± 2.64	–1 ± 2.83	–2.5 ± 4.9	7 ± 5.66
Albumin	control (*n* = 7)	0 ± 0.71	–0.33 ± 0.58	-	-	-	-
	fucoidan (*n* = 13)	0.29 ± 0.49	0 ± 0	0 ± 0	0 ± 0	0 ± 0	–0.5 ± 0.71
Pre-albumin	control (*n* = 7)	–0.86 ± 6.81	–11.05 ± 4.03	-	-	-	-
	fucoidan (*n* = 13)	3.19 ± 8.98	–0.28 ± 8.05	–3.05 ± 3.46	-	–2.65 ± 2.33	6.95 ± 4.03
Transferrin	control (*n* = 7)	23.12 ± 88.39	–34 ± 41.79	-	-	-	-
	fucoidan (*n* = 13)	29.92 ± 30.15	9 ± 7.45	-	-	-	-
T-Protein	control (*n* = 7)	–0.6 ± 1.14	1 ± 1	-	-	-	-
	fucoidan (*n* = 13)	0.29 ± 0.48	0.33 ± 0.51	0.5 ± 0.71	0 ± 1.41	0.5 ± 0.71	0 ± 1.41
GOT (U/L)	control (*n* = 7)	3.6 ± 8.73	5.33 ± 13.05	-	-	-	-
	fucoidan (*n* = 13)	–1.86 ± 9.74	–4.67 ± 10.5	–4.33 ± 13.86	–7.5 ± 9.19	17 ± 21.92	–4.5 ± 2.12
GPT (U/L)	control (*n* = 7)	4.6 ± 8.65	4 ± 8.18	-	-	-	-
	fucoidan (*n* = 13)	1.57 ± 17.71	–2.7 ± 11.83	–5.67 ± 7.76	–7.5 ± 2.12	6 ± 18.38	–5 ± 9.89
BUN (mg/dl)	control (*n* = 7)	–5 ± 2.5	–4.67 ± 6.65	-	-	-	-
	fucoidan (*n* = 13)	2 ± 5.47	3.67 ± 6.5	–1.67 ± 2.08	2.5 ± 7.77	–1.5 ± 0.7	-
Creatinine (mg/dl)	control (*n* = 7)	–0.04 ± 0.16	–0.08 ± 0.235	-	-	-	-
	fucoidan (*n* = 13)	0.06 ± 0.08	0 ± 0.13	0.053 ± 0.098	–0.01 ± 0.16	–0.02 ± 0.233	–O.U ± 2.32
TG (mg/dl)	control (*n* = 7)	-	-	-	-	-	-
	fucoidan (*n* = 13)	-	–8.4 ± 94.13	–89.25 ± 80	-	–82.5 ± 136.47	–95.5 ± 147.78
Choloesterol(mg/dl)	control (*n* = 7)	-	-	61 ± 79. 19	-	-	-
	fucoidan (*n* = 13)	-	–8.8 ± 20.6	–9.5 ± 13.43	-	-	–12.5 ± 57.27

WBC = White blood cell; NLR = neutrophil-to-lymphocyte ratio; GOT = aspartate aminotransferase; GPT = alanine aminotransferase; BUN = blood urea nitrogen; TG = Triglyceride; E = during follow period.

∆: The changed concentrations of cytokines (supplementation week – 0 week).

### Oligo-fucoidan could improve survival rate and quality of life

The survival rates of the subjects in the control and fucoidan groups were 20 and 28.6%, respectively, during the study period. The overall quality of life was improved in the fucoidan group during the study period. This improvement was higher than that observed in the control group; however, the difference was not statistically significant. In terms of pain, dyspnoea, and insomnia, decreasing and increasing trends were noted during the study period in the fucoidan and the control group (Tables 4 and 5).

**Table 4:** Tine EORTC QI Q C30 scores of subjects

Variables	Groups (number)	Baseline	8th week	20th week	36th week	44th week	E-12th week	E-24th week
Physical function	control (*n* = 7)	1.71 ± 0.95	2.2 ± 1.3	2.3 ± 0.6	-	-	-	-
	fucoidan (*n* = 13)	2 ± 1.1	1.9 ± 1.1	22 ± 1.1	1.0 ± 0	15 ± 0.7	1.5 ± 0.7	2.0 ± 0
Role function	control (*n* = 7)	1.6 ± 1.1	1.6 ± 0.9	1.6 ± 0.6	-	-	-	-
	fucoidan (*n* = 13)	1.9 ± 1.1	2.0 ± 1	2.0 ± 1.2	1.5 ± 0.7	1.5 ± 0.7	1.5 ± 0.7	2.0 ± 1.4
Emotional function	control (*n* = 7)	1.7 ± 0.8	2.2 ± 0.8	2.0 ± 0	-	-	-	-
	fucoidan (*n* = 13)	1.9 ± 0.8	1.3 ± 0.5	1.6 ± 0.9	1.5 ± 0.7	2.0 ± 1.4	2.0 ± 1.4	2.5 ± 0.71
Cognitive function	control (*n* = 7)	1.6 ± 0.8	2.2 ± 0.5	2.0 ± 0	-	-	-	-
	fucoidan (*n* = 13)	2.1 ± 1.0	1.4 ± 0.5	1.8 ± 0.5	1.5 ± 0.7	1.5 ± 0.7	1.5 ± 0.7	2.0 ± 0
Social function	control (*n* = 7)	1.6 ± 0.8	2.6 ± 0.9	1.6 ± 0.6	-	-	-	-
	fucoidan (*n* = 13)	2.1 ± 1.1	1.6 ± 1.1	2.2 ± 1.3	1.0 ± 0	2.0 ± 1.4	2.0 ± 1.4	2.5 ± 0.7
Overall quality of life	control (*n* = 7)	4.4 ± 1.5	3.8 ± 0.8	3.3 ± 1.2	-	-	-	-
	fucoidan (*n* = 13)	3.9 ± 2.1	4.4 ± 1.3	4.4 ± 13	5.5 ± 0.7	5.0 ± 0	45 ± 0.7	3 ± 1.4
Fatigue	control (*n* = 7)	2.1 ± 0.7	2.4 ± 1.1	2.7 ± 0.6	-	-	-	-
	fucoidan (*n* = 13)	2.4 ± 1.0	2.3 ± 1.0	2.6 ± 0.9	2.0 ± 0	2.0 ± 0	2.0 ± 0	2.5 ± 0.7
Nausea and vomiting	control (*n* = 7)	1.6 ± 0.5	1.4 ± 0.6	20 ± 1.0	-	-	-	-
	fucoidan (*n* = 13)	1.5 ± 1.0	1.4 ± 0.5	1.4 ± 0.6	-	-	-	-
Paiu	control (n = 7)	2.1 ± 0.7	2.0 ± 0	2.0 ± 0	-	-	-	-
	fucoidan (n = 13)	2.2 ± 1.0	1.7 ± 0.8	2.0 ± 1.2	1.5 ± 0.7	1.5 ± 0.7	2.0 ± 0	2.0 ± 0
Dyspnea	control (n = 7)	1.9 ± 0.9	2.2 ± 1.1	3.0 ± 1.0	-	-	-	-
	fucoidan (n = 13)	2.2 ± 1.0	2.1 ± 1.4	1.8 ± 0.8	1.5 ± 0.7	1.5 ± 0.7	2.0 ± 0	2.0 ± 0
Insomnia	control (n = 7)	1.7 ± 0.8	2.2 ± 0.5	2.0 ± 0	-	-	-	-
	fucoidan (n = 13)	2.1 ± 1.1	1.7 ± 1.1	1.8 ± 1.1	1.5 ± 0.71	-	1.5 ± 0.7	2.0 ± 0
Loss of appetite	control (n = 7)	1.7 ± 1.1	1.6 ± 0.6	2.0 ± 1.0	-	-	-	-
	fucoidan (n = 13)	2.3 ± 1.0	1.6 ± 0.8	1.0 ± 1.6	l.8 ± 0.7	2.8 ± 0.7	2.0 ± 0	2.0 ± 0
Constipation	control (n = 7)	1.6 ± 0.5	2.0 ± 0	2.0 ± 0	-	-	-	-
	fucoidan (n = 13)	1.8 ± 0.8	1.4 ± 1.1	1.8 ± 1.2	1.0 ± 0	l.8 ± 0.7	l.8 ± 0.7	2.0 ± 0
Diarrhea	control (n = 7)	1.4 ± 0.5	1.8 ± 0.5	1.3 ± 0.6	-	-	-	-
	fucoidan (n = 13)	1.5 ± 1.0	1.3 ± 0.5	1.6 ± 0.6	2.0 ± 0	2.0 ± 0	2.0 ± 1.4	2.0 ± 0.7
Financial difficulties	control (n = 7)	1.7 ± 0.8	1.6 ± 0.9	1.7 ± 0.6	-	-	-	-
	fucoidan (n = 13)	2.0 ± 1.1	1.6 ± 0.5	2.4 ± 1.1	2 ± 1.4	2 ± 1.4	2 ± 1.4	2.0 ± 0

The EORTC QLQ-C30 questionnaire: a Quality-of-Life questionnaire is designed to measure cancer patients’ physical, psychological, and social functions.

**Table 5:** The changed EORTC QLQ C30 scores in the two groups during supplementation period

Variables	Groups (number)	Δ8th week	Δ20th week	Δ36th week	Δ44th week	ΔE-12th week	ΔE-24th week
Physical function	control (*n* = 7)	0.6 ± 0.54	0.33 ± 0.57	-	-	-	-
	fucoidan (*n* = 13)	0 ± 0	–0.17 ± 0.4	0 ± 0	0 ± 0	0.5 ± 0.7	0.5 ± 0.7
Role function	control (*n* = 7)	0.4 ± 0.89	1.33 ± 1.15	-	-	-	-
	fucoidan (*n* = 13)	0 ± 0	0 ± 0	0 ± 0	0 ± 0	0 ± 0	0.5 ± 2.12
Emotional function	control (*n* = 7)	0.8 ± 0.54	0.5 ± 0.43	–1 ± 1.08	-	-	-
	fucoidan (*n* = 13)	–2.5 ± 0.35	–0.5 ± 0.51	–0.93 ± 0.96	0.21 ± 0.29	0.25 ± 0.35	1 ± 0.7
Cognitive function	control (*n* = 7)	1.4 ± 0.54	0 ± 0	-	-	-	-
	fucoidan (*n* = 13)	–0.43 ± 0.97	–0.2 ± 0.44	0 ± 0	0 ± 0	0.5 ± 0.7	1.5 ± 0.7
Social function	control (*n* = 7)	0.2 ± 0.44	0 ± 0	-	-	-	-
	fucoidan (*n* = 13)	–0.14 ± 0.37	0.2 ± 0.44	–0.5 ± 0.7	0.5 ± 0.7	0.5 ± 0.7	1 ± 1.41
Overall quality of life	control (*n* = 7)	–0.6 ± 1.34	–2 ± 2	-	-	-	-
	fucoidan (*n* = 13)	0 ± 1.63	0.2 ± 0.83	–0.5 ± 0.7	–1 ± 0	–1.5 ± 0.7	–3 ± 1.41
Fatigue	control (*n* = 7)	0.43 ± 0.89	0.83 ± 0.6	-	-	-	-
	fucoidan (*n* = 13)	0.28 ± 0.4	0.53 ± 0.3	0 ± 0.47	0.5 ± 0.24	0.33 ± 0	1 ± 0.94
Nausea and vomiting	control (*n* = 7)	0 ± 0	1 ± 1	-	-	-	-
	fucoidan (*n* = 13)	0.14 ± 0.37	0 ± 0.7	0 ± 0	1 ± 1.41	0.5 ± 0.7	0.5 ± 0.7
Pain	control (*n* = 7)	0.2 ± 0.44	0.33 ± 0.57	-	-	-	-
	fucoidan (*n* = 13)	0 ± 0	0.2 ± 0.44	0 ± 0	0 ± 0	0 ± 0	1 ± 0
Dyspnea	control (*n* = 7)	0.4 ± 1.14	1.33 ± 1.15	-	-	-	-
	fucoidan (*n* = 13)	0.43 ± 0.97	0 ± 0	0 ± 0	0 ± 0	0.5 ± 0.7	05 ± 0.7
Insomnia	control (*n* = 7)	0.6 ± 0.54	0.5 ± 0.7	–0.5 ± 2.12	-	-	-
	fucoidan (*n* = 13)	0.14 ± 0.37	0.2 ± 0.44	–1 ± 1.41	-	–3 ± 1.41	–0.5 ± 0.7
Loss of appetite	control (*n* = 7)	0.2 ± 0.44	1 ± 1	-	-	-	-
	fucoidan (*n* = 13)	–0.29 ± 0.95	0 ± 0.7	0 ± 0	1 ± 1.41	0.5 ± 0.7	05 ± 0.7
Constipation	control (*n* = 7)	0.4 ± 0.54	0.67 ± 0.57	-	-	-	-
	fucoidan (*n* = 13)	0 ± 1	0.4 ± 0.89	0 ± 0	0.5 ± 0.7	0.5 ± 0.7	1 ± 0
Diarrhea	control (*n* = 7)	0.4 ± 0.54	0 ± 0	-	-	-	-
	fucoidan (*n* = 13)	0.14 ± 0.37	0.4 ± 0.54	0.5 ± 0.7	0.5 ± 0.7	0.5 ± 2.12	1 ± 0
Financial difficulties	control (*n* = 7)	0 ± 0.7	0.33 ± 0.57	-	-	-	-
	fucoidan (*n* = 13)	–0.29 ± 0.75	0.2 ± 0.44	0.5 ± 0.7	0.5 ± 0.7	0.5 ± 0.7	0.5 ± 0.7

∆: The changed concentrations of cytokines (supplementation week – 0 week).

### Oligo-fucoidan could increase CD19 population

The proportions of immune cell types in the two groups during the supplementation period are shown in Table 6. The percentages of CD3CD4, CD3CD8, CD4CD25, CD4CD45RO, and CD4CD45RA were not significantly different between the two groups, and no trend of increase or decrease was found in individual groups, except for CD19, during the study period (Tables 6 and 7). In the fucoidan group, the percentage of CD19 cells at the 44th week during the supplementation period and the E-12th week during the supplementation period was higher than that at baseline (*P* < 0.05).

**Table 6:** The proportion of immune cell types in the two groups during the supplementation period.

Variables	Groups (number)	Baseline	8th week	20th week	36th week	44th week	E-12th week	E-24th week
CD3CD4 (%)	control (*n* = 7)	30.9 ± 14.1	35.0 ± 11.5	31–7 ± 14.2	-	-	-	-
	fucoidan (*n* = 13)	27.2 ± 8.3	25.3 ± 17.1	26.2 ± 11.5	23.0 ± 15.6	11.0 ± 5.7	20.0 ± 14.1	11.5 ± 7.8
	control (*n* = 7)	17.7 ± 5.6	24.2 ± 5.1	20.7 ± 6.7	-	-	-	-
CD3CD8 (%)	fucoidan (*n* = 13)	15.2 ± 5.6	14.6 ± 10.9	11.0 ± 6.9	22.5 ± 9.2	14.0 ± 11.3	17.5 ± 7.8	19.0 ± 12.7
	control (*n* = 7)	0.6 ± 0.8	0.8 ± 0.5	1 0 ± 1.0	-	-	-	-
CD4CD25 (%)	fucoidan (*n* = 13)	0.8 ± 0.9	0.7 ± 0.8	1.0 ± 0.6	0.5 ± 0.7	1.0 ± 0	0.5 ± 0.7	1.0 ± 0
	control (*n* = 7)	11.0 ± l 3.9	2.6 ± 1.7	9.7 ± 9.9	-	-	-	-
CD4CD45RA (%)	fucoidan (*n* = 13)	8.3 ± 11.9	8.3 ± 9.3	11.0 ± 12.1	0.5 ± 0.7	2.0 ± 1.4	2.5 ± 2.1	0.5 ± 0.7
	control (*n* = 7)	1.7 ± 1.5	0.8 ± 0.8	1.7 ± 1.2	-	-	-	-
CD4CD45RO (%)	fucoidan (*n* = 3)	1.2 ± 1.6	2.1 ± 1.2	2.2 ± 1.7	1.0 ± 1.4	1.5 ± 0.7	-	11 ± .41
	control (*n* = 7)	10.1 ± 6.4	6.2 ± 2.9	13.7 ± 9.3	-	-	-	-
CD19 (%)	fucoidan (*n* = 13)	11.6 ± 5.5	11.1 ± 5.4	15.2 ± 7.7	21.0 ± 11.3	27.5 ± 6.4*	21.5 ± 3.5*	18 ± 5.66

∆: The data 44th week and E-12th week compared with baseline had significant different (*P* < 0.05).

**Table 7:** The changed proportion of immune cell types in the two groups during supplementation period

Variables	Groups (number)	Δ8th week	Δ20th week	Δ36th week	Δ44th week	ΔE-12th week	ΔE-24th week
CD3CD4 (%)	control (*n* = 7)	–1.6 ± 16.56	–6.67 ± 10.69	-	-	-	-
	fucoidan (*n* = 13)	–6.14 ± 12.06	–2.83 ± 9.45	–51 ± 2.78	–16.5 ± 3.53	–81 ± 1.31	–16.5 ± 4.95
CD3CD8 (%)	control (*n* = 7)	4.8 ± 7.19	1.67 ± 4.72	-	-	-	-
	fucoidan (*n* = 13)	–1.571 ± 4.97	–4.83 ± 6.85	1.5 ± 2.12	–6.5 ± 0.7	–3.5 ± 3.53	–1.5 ± 0.7
CD4CD25 (%)	control (*n* = 7)	–2 ± 0.44	0 ± 1	-	-	-	-
	fucoidan (*n* = 13)	–0.14 ± 0.37	0 ± 0.63	–0.5 ± 0.7	0 ± 0	0 ± 0	0 ± 0
CD4CD45RA (%)	control (*n* = 7)	–10 ± 16.21	–4.33 ± 25.16	-	-	-	-
	fucoidan (*n* = 13)	1.83 ± 10.55	0.6 ± 20.14	-	-	-	-
CD4CD45RO (%)	control (*n* = 7)	–0.8 ± 1.92	–0.33 ± 2.3	-	-	-	-
	fucoidan (*n* = 13)	0.83 ± 1.72	0.4 ± 1.67	-	-	-	-
CD19 (%)	control (*n* = 7)	–3.8 ± 7.66	4.67 ± 15.14	-	-	-	-
	fucoidan (*n* = 13)	0.57 ± 6.26	4.17 ± 6.01	3 ± 11.31	9.5 ± 6.36	4 ± 2.82	0 ± 5.65

∆: The changed concentrations of cytokines (supplementation week – 0 week).

#### The changed concentrations of cytokines (a) IL-1β, (b) IL-6, (c) IL-8 in the two groups during supplementation period

**Fig. 2:**
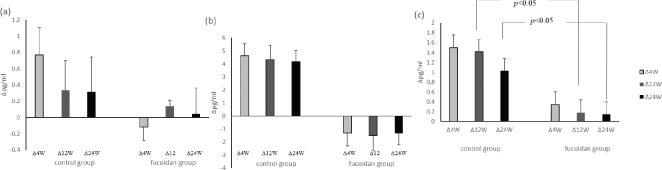
The changed concentrations of cytokines (a) IL-1β, (b) IL-6, (c) IL-8 in the two groups during supplementation period. ∆: The changed concentrations of cytokines (supplementation week – 0 week). *: significant different compared with control group among groups (*P* < 0.05).

Plasma levels of IL-1β, IL-6, and IL-8 were assayed using ELISA kits (Fig. 2). There were no differences between the cytokine levels in the control and fucodian groups. However, the changed concentrations of IL-8 cytokines in the two groups during supplementation period (12W and 24W) (*P* < 0.05).

## Discussion

The 5-year survival rate for individuals diagnosed with lung cancer in the United States has experienced marginal improvement in recent years. The combined 5-year relative survival rate for all types of lung cancer, including both NSCLC and SCLC, stands at 19%. Notably, the 5-year survival rate for NSCLC is comparatively higher at 23%, while SCLC lags significantly behind with a mere 6% survival rate (17). In Taiwan, the 2-year lung cancer survival rate increased by 19.81% (95% CI 14.90%–24.71%) after the introduction of epidermal growth factor receptor (EGFR) mutations (18). In this study, among subjects with disease stages beyond IIIB, the control group exhibited a 2-year survival rate of 20%, whereas the fucoidan group showed a higher 2-year survival rate of 28.6% throughout the study duration. The survival rate in the control group (20%) was similar to that in other studies (19.81%), and the survival rate in the fucoidan group (28.6%) was higher than that in other studies (19.81%), indicating that supplementation with fucoidan during conventional treatment can help improve the 2-year survival rate.

Patients are prone to physical changes and complications due to the disease and various treatments, which will affect the course of the disease and the efficacy of treatment. Therefore, one of the goals of treatment is to improve the quality of life of the patients. The EORTC QLQ-C30 includes 30 questions that measure five function scales, three symptom scales, five single symptom items, and financial difficulty (19–21). For all scales that measure function, a higher score represents a better quality of life, and for all symptom scales/items, a lower score indicates a better quality of life. According to other studies, the quality of life of patients with lung cancer is worse than that of patients with other cancers in all categories (22). Similar results have been reported in Taiwan (23). Compared with other cancer patients, lung cancer patients have a poorer quality of life and lower survival rates (24). The quality of life of patients with lung cancer gradually declines as the disease progresses and remains poor when receiving treatment, particularly due to pain, loss of appetite, nausea, nervousness, difficulty falling asleep, and depression (25–28). In terms of lung cancer severity, patients with more severe lung cancer stages have poorer quality of life (23, 24). During the study, an enhancement in the quality of life among patients in the fucoidan group was observed, surpassing that of the control group. However, it’s worth noting that this difference did not reach statistical significance. Pain, dyspnoea, and insomnia showed a decreasing trend in the fucoidan group and an increasing trend in the control group during the study period.

Immunological analysis of the tumour microenvironment has shown the potential to improve prognosis and predict response to immunotherapy. Studies have indicated the potential for improved outcomes in ovarian cancer, colorectal cancer, breast cancer, and lung cancer (29–33), and that immunological parameters can better predict clinical outcomes and the effectiveness of treatment (34). Immune cells related to lung cancer include CD4 + T cells, CD8 + T cells, granulocytes, monocytes, B cells, and NK cells (35). B cells are good indicators of clinical outcomes (36, 37). In addition to secreting immunoglobulins, B cells play a role in antigen presentation, cytokine production, lymphoid organogenesis regulation, effector T cell differentiation, and dendritic cell function (38). The pivotal role of B cells in numerous immune-mediated disorders has been substantiated through signalling pathways involving B cell receptors and functionally related cell surface receptors, including CD19, CD21, CD22, CD40, B cell- activating factor receptor, and Fcγ receptor IIb. Hence, modulation of these receptors may be a potential therapeutic approach (39). In this study, we also found that the percentage of CD19 at 44th week during the supplementation period and E-12th week during the supplementation period in the fucoidan group was higher than that at baseline (*P* < 0.05). This finding warrants further investigation.

NLR is a strong prognostic marker for NSCLC (40). Another study indicated that an elevated NLR is associated with a worse survival rate (41). In this study, NLR tended to be lower in the fucoidan group than in the control group; however, the difference was not statistically significant. This finding warrants further investigation.

## Conclusions

Oilgo-fucoidan may be a potential adjuvant therapy for improving the survival rate, quality of life, and immunity of patients with lung cancer.

## Data Availability

The data that support the findings of this study are available from the corresponding author, upon reasonable request.

## Declarations

Author Contributions: methodology, Chia-Ju Shih; validation, Ya-ling Chiou.; formal analysis, Chia-Ju Shih, Ya-ling Chiou; investigation, Chia-Ju Shih, Ya-ling Chiou; resources, Tu-Chen Liu.; data curation, Chia-Ju Shih, Ya-ling Chiou; writing-original draft preparation, Ya-ling Chiou; writing-review and editing, Ya-ling Chiou.; visualization, Ya-ling Chiou.; supervision, Tu-Chen Liu.; project administration, Chia-Ju Shih.; funding acquisition, Ya-ling Chiou. All authors have read and agreed to the published version of the manuscript.
